# Rapid Assessment of Surface Markers on Cancer Cells Using Immuno-Magnetic Separation and Multi-frequency Impedance Cytometry for Targeted Therapy

**DOI:** 10.1038/s41598-020-57540-7

**Published:** 2020-02-20

**Authors:** Zhongtian Lin, Siang-Yo Lin, Pengfei Xie, Chen-Yong Lin, Gulam M. Rather, Joseph R. Bertino, Mehdi Javanmard

**Affiliations:** 1 Rutgers University New Brunswick; Department of Electrical and Computer Engineering, 94 Brett Rd, New Brunswick, NJ 08854 USA; 2 Rutgers Cancer Institute of New Jersey, Rutgers University, 195 Little Albany St, New Brunswick, NJ 08901 USA; 3 Georgetown University, School of Medicine, 3900 Reservoir Rd NW, Washington, DC 20007 USA

**Keywords:** Biosensors, Microfluidics

## Abstract

The rapid qualitative assessment of surface markers on cancer cells can allow for point-of-care prediction of patient response to various cancer drugs. Preclinical studies targeting cells with an antibody to “activated” matriptase conjugated to a potent toxin show promise as a selective treatment for a variety of solid tumors. In this paper, we implemented a novel technique for electrical detection of proteins on surfaces of cancer cells using multi-frequency microfluidic impedance cytometry. The biosensor, consists of two gold microelectrodes on a glass substrate embedded in a PDMS microfluidic channel, is used in conjugation with immuno-magnetic separation of cancer cells, and is capable of differentiating between bare magnetic beads, cancer cells and bead-cell aggregates based on their various impedance and frequency responses. We demonstrated proof-of-concept based on detection of “activated” matriptase proteins on the surface of cultured Mantle cells.

## Introduction

Survival of patients with cancer has been significantly improved due to the developments in new therapeutics for patients in the past decade, however, once metastatic, the disease remains incurable. Thus, new therapeutic agents as well as diagnostic tools predicting patient response are urgently needed. Overexpression of matriptase (a membrane-bound serine type II protease) has been found in various epithelial tumor and blood malignancies suggesting that the enzyme can be used as marker for CTC detection. Due to the expression of “activated” matriptase in Mantle cells, anti-matriptase monoclonal antibody (M69) conjugated to monomethyl auristatin E (MMAE) for selectively targeting Mantle cells has been demonstrated as a promising therapeutic with high levels of efficacy with minimal potential side effects^1,2^.

One of the key processes playing a role in modulation of the tumor environment is (membrane-anchored) proteolysis^3^. Matriptase, a type II transmembrane serine protease, is an important pericellular protease that has an effect on tumor microenvironments, as it is responsible for initiating the protease cascade and activating growth factors. Matriptase is broadly expressed in epithelial tissues^4^, where the enzyme plays a crucial role in forming and maintaining epithelium integrity and epidermal differentiation, and also the placenta development, to give a few examples. There is growing evidence showing that altered matriptase expression is potentially important in hematological cells and also neoplasms^5^. Matriptase is shown to be expressed on the surface of THP-1 human monocytic cells^5^. Matriptase has also been detected on the surfaces of a wide range of cells including peritoneal macrophages^6^, two Burkitt lymphoma (BL) cells, and also human leukemia and chronic lymphocytic leukemia^7,8^. In contrast to the situation in epithelial/carcinoma cells, these hematological cells express no or low levels of HAI-1 (Hepatocyte Growth Factor Activator Inhibitor Type 1). Various studies have examined the role and regulation of matriptase in human B-cell lymphomas, and data shows that it is expressed in various non-Hodgkin B-cell lymphomas with implication for tumor behavior^9^. Given the importance of matriptase in tumor behavior and its expression on a wide variety of tumor cell types, the targeted delivery of cancer drugs to the tumor site shows great promise for enhancing drug efficacy and minimizing toxicity towards non-cancerous cells. Thus, the ability to rapidly isolate tumor cells in blood and qualitatively assess matriptase surface expression levels using inexpensive miniaturized instrumentation can provide guidance and great insights in further developing this novel and promising therapeutic approach. We emphasize here that activated matriptase is present in most but not all epithelial cancers, and therefore the M-69 (anti-matriptase) antibody can identify those tumors that express activated matriptase alone or complexed with its inhibitor Hepatocyte Growth Factor Activator Inhibitor Type 1 (HAI-1).

Current technologies for sorting and assessment of surface markers on cells are bulky and unsuitable for point-of-use analysis and deployment in large-scale clinical studies. Fluorescence cytometry (FCM) and fluorescence activated cell sorting (FACS) are the gold standards for high-throughput rapid cell sorting and surface marker analysis. The technology is, however, bulky and expensive and thus not suitable for point-of-care use.

The gold standard technology for cancer cell isolation and marker assessment is the CellSearch CTC (Circulating Tumor Cells) Test, which uses magnetic bead based pre-concentration and fluorescent tagging of the cells and fluorescently analyzing the cell surfaces^10^. Various configurations of the CTC chip, developed by Toner and colleagues, utilizes optimal microfluidic geometries for highly efficient immuno-separation of CTCs from whole blood based on using EpCAM (epithelial cell adhesion molecule) capture antibodies^11,12^. More recently, the MagSweeper, an immuno-magnetic separation technique has been developed which is able to enrich tumor cells from blood by 108-fold and can process 9 ml of blood per hour^12^. While magnetic immuno-separation methods are advantageous in that they allow for highly efficient enrichment of rare cells, yet one of primary drawbacks is that once the cells have been tagged with magnetic particles, it is difficult to separate the bead-cell aggregates from the mixture of bare magnetic beads, since magnetic fields will attract both. Thus, in order to separate the beads from the bead-cell aggregates, a trained biologist has to view the mixture visually under a microscope and manually pick the bead-cell aggregates off the slides. Alternatively, this can be done using bulky robotics with automated image analysis capability, or by staining the cells and using fluorescence analysis. All of the above technologies utilize optical-based detection, which requires bulky instrumentation and is thus unsuitable for point-of-care analysis. Previously, Holmes *et al*. demonstrated an impedance labelling method for identifying target antigen expressing cell subpopulations from a heterogeneous mixture^13^. Most recently, Lee and colleagues developed a promising microfluidic electronic sensing platform, which involves tagging CTCs with magnetic nanoparticles and using a “μHall Detector” to detect the MNP (magnetic nano-particles) tagged CTCs but not the MNPs, since the individual MNPs are too small to accumulate sufficient signal to trigger a response in the μHall Detector, and thus the sensor only responds to the MNP tagged CTCs^14^.

Various promising technologies have also been developed making use of label-free tumor cell separation based on physical properties such as size^15–18^ and dielectric permittivity^19–21^, allowing for downstream molecular analysis of the isolated cells. Although there is great interest in developing technologies that can be used for analysing circulating tumor cells, the ability to analyse dissociated cancer cells obtained from a tissue biopsy is also of great importance in predicting patient response to targeted therapies.

Here, we used Maver cell, a Mantle cell line, as a prototype to test the feasibility of a technique that makes use of immune-magnetic separation to pre-concentrate cancer cells and electrical impedance detection to differentiate between isolated cells and bare magnetic beads to assess matriptase expression on cancer cells. We envision ultimately using this technique to isolate tumor cells from complex samples (like dissociated cells obtained from a tissue biopsy) to predict cancer patient response to novel targeted therapeutics using anti-neoplastic agents conjugated with anti-matriptase antibody. We emphasize that our proposed technique can be used in conjunction with the above mentioned immuno-magnetic based cancer cell separation techniques to either characterize matriptase levels on tumor cells obtained from a biopsy and then dissociated into cell suspension or circulating tumor cells directly from blood.

**Figure 1:**
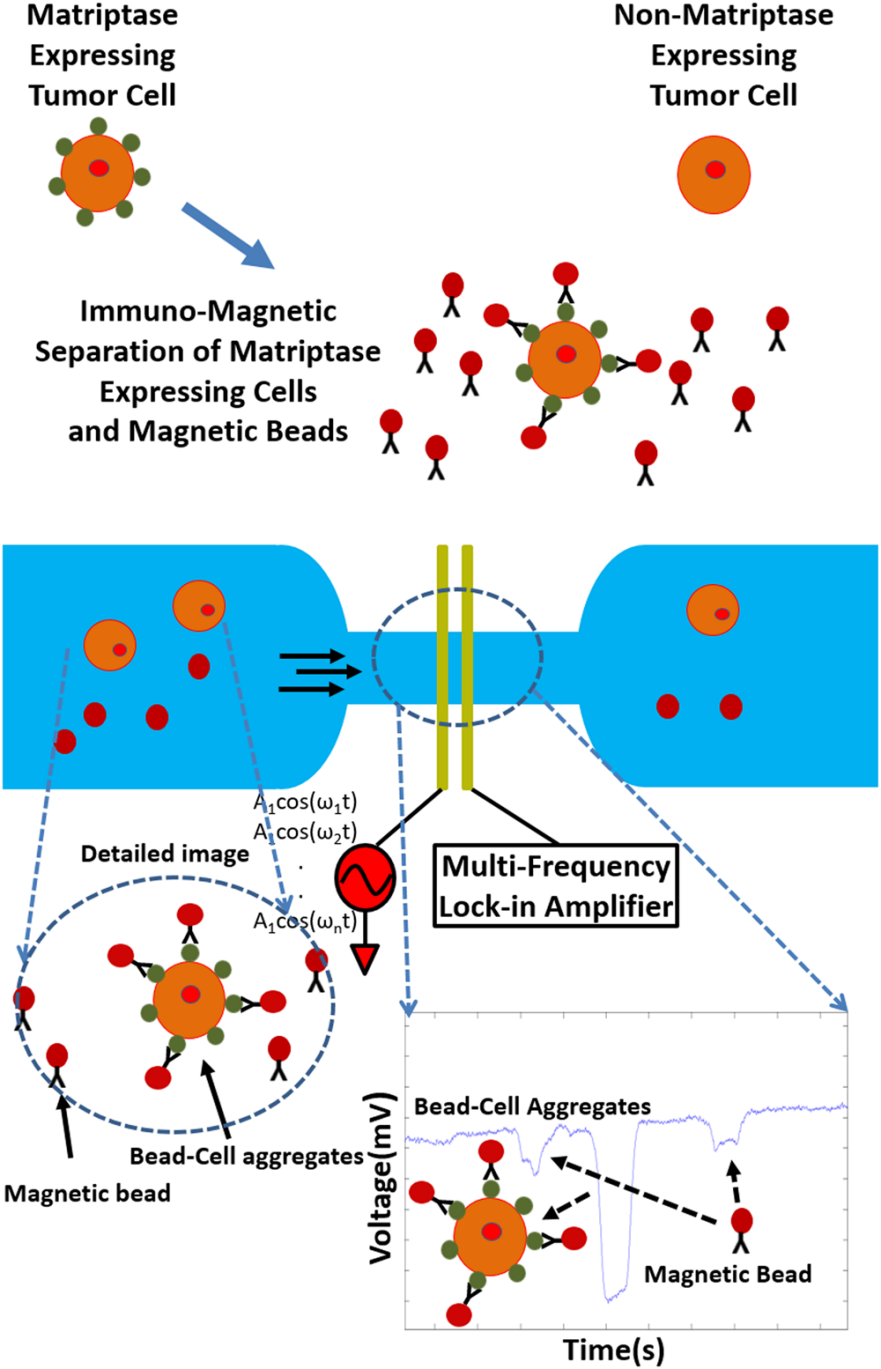
Schematic of biochip. The presence of matriptase expressing cultured cancer cells in the antibody coated beads mixture results in beads binding to the cell and aggregating. Impedance based sizing allows differentiation between magnetic beads and bead-CTC aggregates.

**Figure 2:**
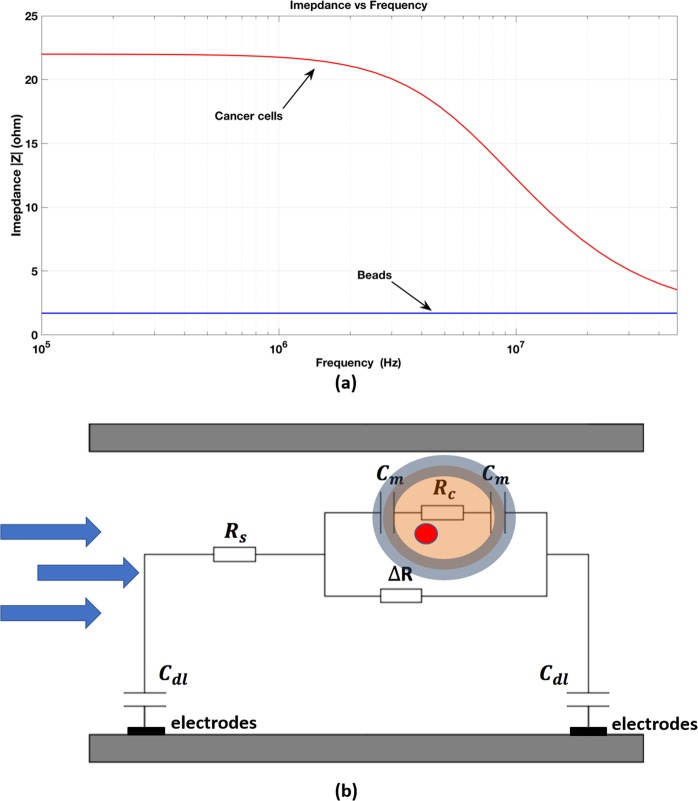
(**a**) Theoretical model for the impedance change for cells and beads as a function of frequency when beads or cells pass over sensor. (**b**) The electrical equivalent circuit model of a two electrode pair system with a cell suspended in buffer. C_dl_: double layer capacitance, R_s_: solution resistance, ΔR: representing the occlusion of ions passing between the electrodes due to the cell volume, C_m_: membrane capacitance, R_c_: cytoplasm resistance.

The basic device is shown in Fig. 1a. Magnetic beads, coated with an anti-matriptase monoclonal antibody (M69) that recognizes “activated” matriptase, are mixed with test sample containing target Mantle cells. The expression of matriptase on the membrane of cancer cells results in bead-cell aggregation. Immuno-magnetic separation is used to extract the magnetic beads and the bead-bound cancer cells from the test sample. The use of multi-frequency electrical impedance cytometry allows for differentiating between unbound beads, non-target cells and bead-cell aggregates. Beads have a relatively flat impedance response with frequency (Fig. 2a), whereas cells exhibit a drop in impedance change as frequency increases (Fig. 2a). This method can be used for detection and qualitative assessment of surface membrane bound protein (i.e. matriptase) levels, as the size and quantity of peaks corresponding to bead-cell aggregates is proportional to concentration of matriptase expressed on the cancer cells. Previously, we demonstrated the feasibility of using digital electronic detection of protein biomarkers^22^ and also the ability to fully miniaturize the footprint of the readout instrumentation to portable and wearable platforms^23^,^23^. We also demonstrated electronic bead aggregate sizing for protein biomarker detection and quantification of soluble proteins^25^. We also demonstrated label-free classification of drug sensitive cells using multi-frequency impedance cytometry in conjunction with supervised machine learning^26^. Here, we utilize information attained by simultaneous measurement of peak intensity at multiple frequencies, which allows effective differentiation between bare beads, and bead-cell aggregates, allowing for detection apparatus to be integrated onto a portable platform.

## Materials and Methods

### Modeling

We model the particle inside the channel and the electrode/electrolyte interface using the circuit model shown in Fig. 2b. We consider a cell to consist of a membrane, which we represent as a capacitor (C_m_) in series with the cytoplasm resistance (R_c_). Using inert electrodes (gold), we assume an ideal polarizable electrode model with no charge transfer resistance at the interface. The model consists of a double layer capacitance (C_dl_) from the left electrode in series with the solution resistance (R_s_) in series with a network of impedance components representing the cell/particle in series with the double layer capacitance (C_dl_) of the right electrode. The impedance of the cell consists of a resistor (ΔR) representing the occlusion of ions passing between the electrodes due to the cell volume in parallel to the membrane capacitance (C_m_) and cytoplasm resistance (R_c_). Equation 1 describes the total impedance across the electrodes.

1 $$Z=\frac{2}{j\omega {C}_{dl}}+{R}_{s}+\Delta R\parallel (\frac{2}{j\omega {C}_{m}}+{R}_{c})$$

The resistive component (ΔR) is dependent on the volume of the cell/particle. Thus volume alone may be difficult to differentiate between cells and beads. However, at frequencies greater than 1 MHz, as membrane capacitance of cells is significantly larger compared to beads, it results in a smaller peak amplitude compared to lower frequencies (f < 1 MHz) which is dependent on cell/particle size. For a bead, one can assume a negligible membrane capacitance (C_m_) and a significantly higher resistivity (R_c_) compared to cells.

### Experiments

**Figure 3:**
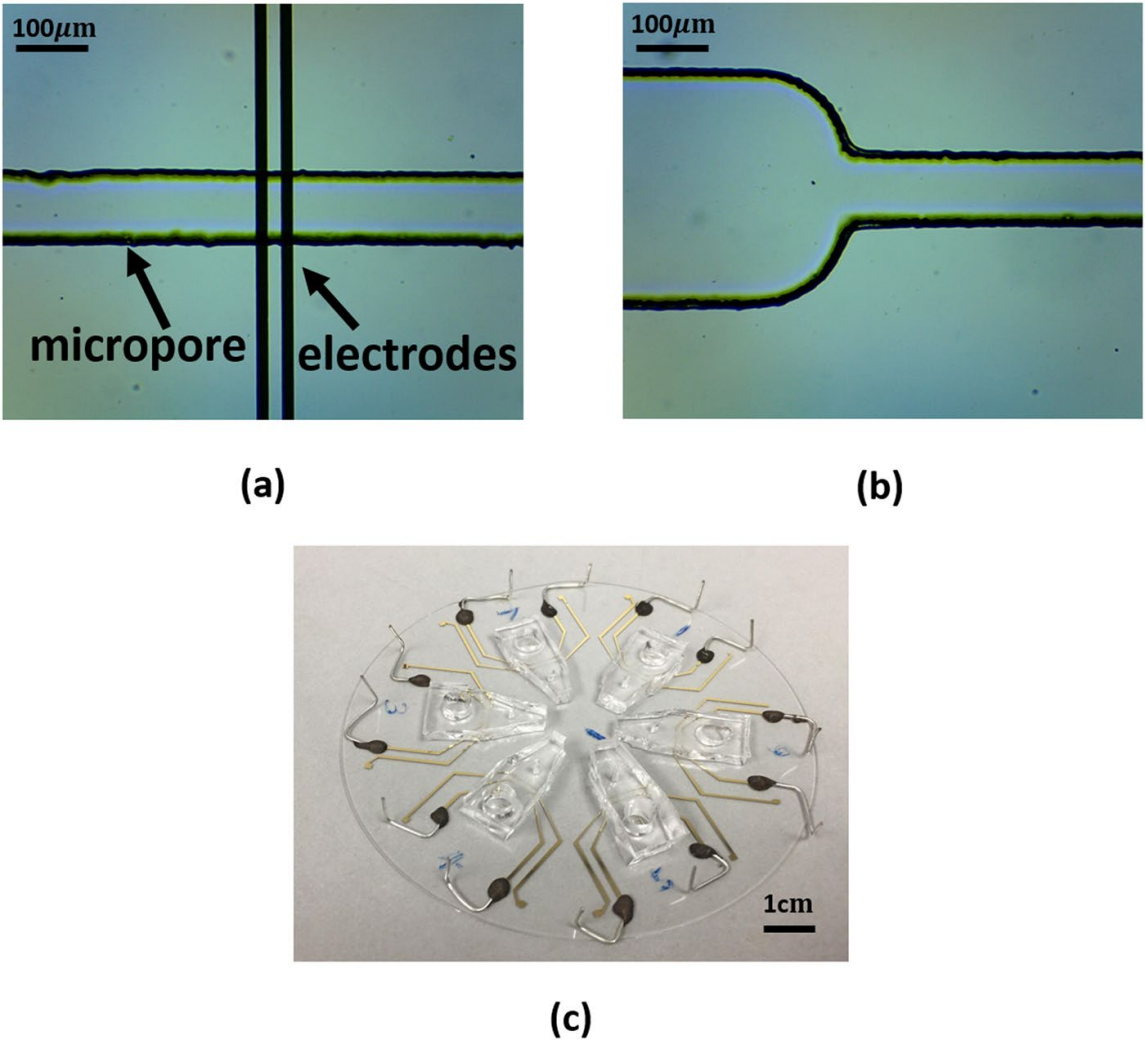
(**a**) Image of the Microfabricated tapered channel entrance into micropore. (**b**) Microfabricated electrodes embedded in channel. (**c**) 6 Microfabricated devices on a 3 inch glass wafer.

**Figure 4:**
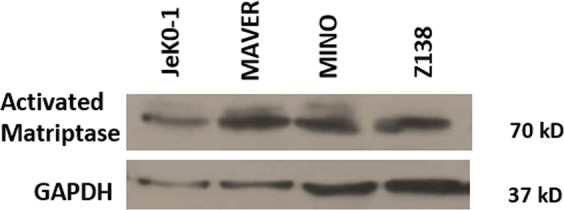
Western Blotting showing the activated matriptase expression in Mantle Cell lymphoma cells (Jek0-1, MAVER, MINO & Z138)^31^. Equal amount of protein was loaded in 10% SDS-PAGE. All blots are from same gel.

The microfabricated biochip (Fig. 3a–c) consists of two gold microelectrodes on a glass substrate with the channel above it formed in a PDMS cover. The micro-channels are 400 µm wide and 20 µm high tapering down to a sensing pore which is 100 µm wide and 20 µm high. The smaller cross sectional area of the sensing pore allows for both focusing of particles and also higher electrical sensitivity to the extent where 2.8 µm beads can be detected at the single bead level. The spacing between the two electrodes is 20 µm and the width of each electrode is 15 µm. The PDMS devices were treated with oxygen plasma to render the surfaces hydrophilic. To begin the proof-of-concept study of CTC detection by using anti-activated matriptse antibody, we used Maver cells as a prototype. Unlike epithelial cancer cells, Maver cells are a subtype B-cell lymphoma called Mantle cell lymphoma, that are cultured in suspension. As such Maver cells are readily available for the magnetic bead-binding experiment, without the additional step to detach cultured epithelial cancer cells leading to degradation of the membrane-bound matriptase. Activated matriptase expression is found in four Mantle cell lines including Maver cells. Maver cells were used to test MFIS (multi-frequency impedance cytometry) and demonstrate that M69 mAb formed aggregates between magnetic beads and cancer cells to generate distinguishable signals. Results of the Western blot analysis demonstrate the presence of a 70-kDa active form of matriptase in those Mantle cell lines using M69, a specific monoclonal antibody to the activated enzyme (Fig. 4). Cancer Cells were suspended in phosphate buffer saline (PBS).

An anti-matriptase monoclonal antibody (M69) conjugated to MMAE selectively targeting Maver Mantle cells was conjugated to 2.8 µm super-paramagnetic sheep anti-mouse IgG beads. The beads were mixed and incubated with cancer cells in PBS containing 0.1% (w/v) BSA for 1 hour, where beads and cancer cells expressing matriptase formed aggregates as visualized optically (Fig. 4). A magnetic separator was used to separate both the bare magnetic beads and the bead-cell aggregates from the mixture. The bare beads and the bead-cell aggregates were resuspended in PBS ready to be analysed using multi-frequency impedance cytometry.

Particles suspended in solution were injected into the micro-channels, where impedance measurements were made at multiple frequencies simultaneously. We performed measurements for three different mixtures. The first was for immune-magnetically separated 2.8 µm magnetic beads in PBS. The second was for a pure suspension of Maver cells in PBS. The third was for a mixture of immuno-magnetically separated magnetic beads and magnetic bead-cell aggregates. For each mixture, the experiment was run for sufficient time until at least several hundred peaks were obtained. The first and third samples were tested three times. A wavelet filter was used for both baseline drift subtraction and also noise reduction, so that peak amplitudes could be readily determined.

### Device fabrication

In order to fabricate the microelectrodes onto a glass wafer, we first designed the photolithography mask using AutoCAD. Then we photo-patterned the image on a 3” glass wafer using standard photolithography. At last, we performed electron beam evaporation to deposit thin film gold electrodes and lift off processing using acetone in an ultrasonicator to fully pattern the electrodes. Photoresist patterning is performed through the procedure below. The glass wafer was cleaned with acetone and methanol, then photoresist was spin coated, soft baked, and the wafer was exposed with ultra-violet light through chromium mask. The photoresist was developed and then underwent a hard bake. After photo-patterning, we deposited 100 nm gold on top of a 10 nm layer of chromium which was used for adhesion.

The micro-channels were made of polydimethyl siloxane (PDMS). The master mold for the channel was an inverted image patterned on a 3 inch silicon wafer. We fabricated the master mold using standard photolithography including wafer cleaning, spin-coating of SU-8 (Microchem Inc., Massachusetts, USA) photoresist, soft baking, ultraviolet light exposure, developing, and hard baking. We poured the PDMS mixture with a ratio of 10 to 1 (pre-polymer to curing agent) onto the silicon wafer. We degased the mixture in a vacuum desiccator to remove the bubbles, and cured the mixture in the oven at 80 °C for about an hour.

After curing, we peeled the PDMS slab off of the wafer, punched 2 mm and 1.2 mm holes as the inlet and outlet respectively. We then aligned and bonded the PDMS channel to our micro-electrode chip after a treatment of both substrates with oxygen plasma. At last, we baked the bonded chip at 75 °C for 20 minutes.

### Sample preparation

**Figure 5:**
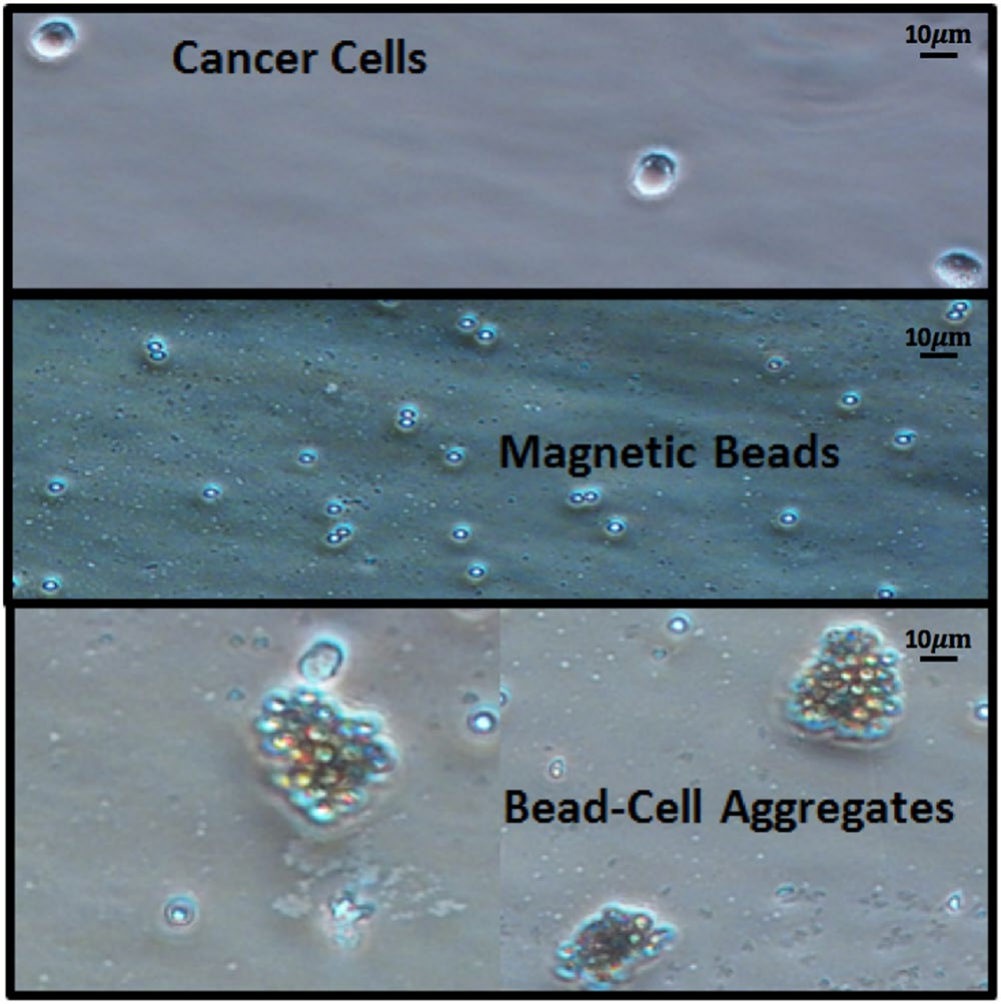
Microscope image of (Top) Cancer Cells, (Middle) Magnetic Beads, and (Bottom) Magnetic Bead-CTC Aggregates.

A monoclonal anti-matriptase antibody (M69) was developed. We used cultured Mantle (Maver cell line) cells (Fig. 3), and 2.8 µm superparamagnetic sheep anti-mouse IgG beads (Life Technology, Carlsbad, CA, USA) (Fig. 3) for the assay. The protocol for immune-magnetic capture of matriptase expressing cancer cells went as follows: Sheep anti-mouse IgG beads were washed with PBS containing 0.1% (w/v) BSA three times. 3 µg of M69 antibody was suspended in PBS. The beads and the antibodies were mixed and rotated for 1 h in an 1.5 ml tube at 25 degrees Celsius to ensure the binding of M69 and the beads. We then washed the beads three times with PBS containing 0.1% (w/v) BSA to remove the excessive antibody. Cultured mantle cells (1.5 million cells/ml) in PBS containing 5% FBS were then added to the 1.5 ml tube and gently rotated for another 1 h at 25 degrees Celsius. Then, we placed the tube in a magnetic separator allowing for extraction of the magnetic beads and bead-cell aggregates (Fig. 5) from the cell suspension followed by washing three times with PBS containing 5% FBS. Finally, the bead-bound cells were suspended in 200 µl PBS containing 0.1% BSA.

### Specificity

The specificity of M69 towards activated matriptase in various cancer cells has been demonstrated by a subset of this manuscript’s co-authors (Lin *et al*.) in the referenced publication^27^. In this report, M69 has been shown to only recognize activated matriptase in complex with the endogenous inhibitor, HAI-1. Whereas, the other anti-matriptase antibody, M32, bound both latent and activated matriptase. Moreover, matriptase is not expressed in blood cells except monocytes^5^. To get rid of the signals derived from monocytes in the blood samples, the interfering noise can be simply eliminated by negative-absorption using the monocytes-specific antibody. We further emphasize that this is a platform technology. Antibody functionalization to beads and passivation to block non-specific cell capture is well established in cellular/molecular assay, and is beyond the scope of this manuscript, which was to demonstrate the ability to qualitatively assess cell surface markers rapidly with electrical detection technology.

### Measurement and data acquisition

In order to extract the electronic signals from the sensor, the biochip was connected to a lock-in amplifier (Zurich Instrument HF2 Series, Zurich Instruments, Zurich, SI) through two wires bonded to the gold pads on the biochip. The amplitude of the input AC voltage was 1 V peak to peak for each channel, and the frequency ranged from 100 KHz to 20 MHz. The gain of the amplifier is 1 kVolt/Ampere. While testing the electrical signal, we monitored the biochip under an optical microscope in order to simultaneously monitor the beads while measuring the electrical signal. The recorded data was then processed using a custom-written Matlab code including wavelet filter for denoising and detrending, and an algorithm for identifying the peaks and quantifying them.

## Results and Discussion

**Figure 6:**
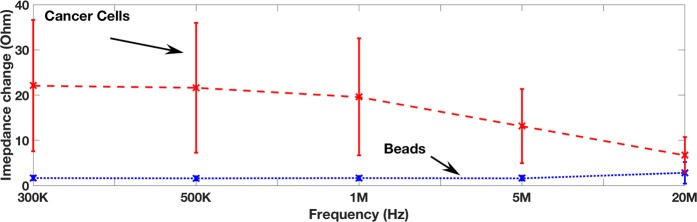
Impedance change as a function of the input AC frequency when a particle passes through the electrodes.

Figure 6 shows the measured particle impedance change figure as a function of frequency over an ensemble of particles. The impedance we plot here is the impedance change when a particle passes through the electrodes. We plot the average impedance change as a function of the frequency at f = 300 KHz, 500 kHz, 1 MHz, 5 MHz and 20 MHz for cells and bare beads. The error bars show the standard deviation for the ensemble of measurements. As seen, cancer cells exhibit a larger peak intensity compared to magnetic beads. The reason for this is because of the larger comparative volume of cells compared to beads. Cells are roughly 2–3X larger in radius compared to beads thus at least 8X larger in volume, which explains why the average impedance change of cells is roughly 8X larger than the beads at low frequencies. As frequency increases, the impedance change of beads remains relatively steady, whereas the impedance change of cells decreases which fits the behaviour expected from our circuit model in Fig. 2a. The impedance starts to decrease at 1 MHz in the sample consisting of purified cancer cells. The reason for which the impedance of the cancer cells drops faster with frequency, as opposed to the bare beads, is because cells exhibit a significantly larger membrane capacitance and smaller membrane resistance compared to bare magnetic beads.

**Figure 7:**
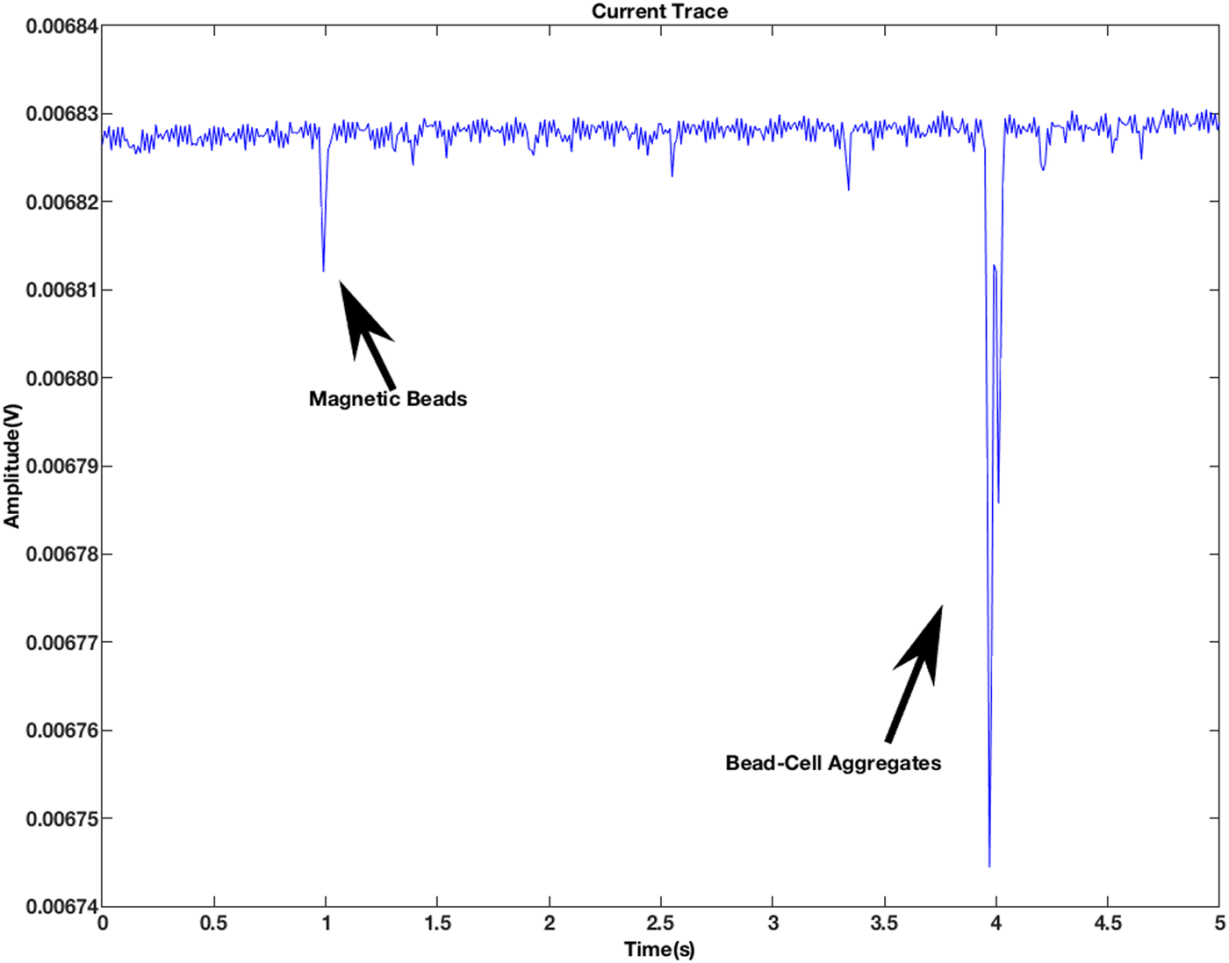
Peaks due to magnetic beads and bead-CTC aggregates in raw data. The frequency and amplitude of the input AC voltage were 500 kHz and 1 V.

**Figure 8:**
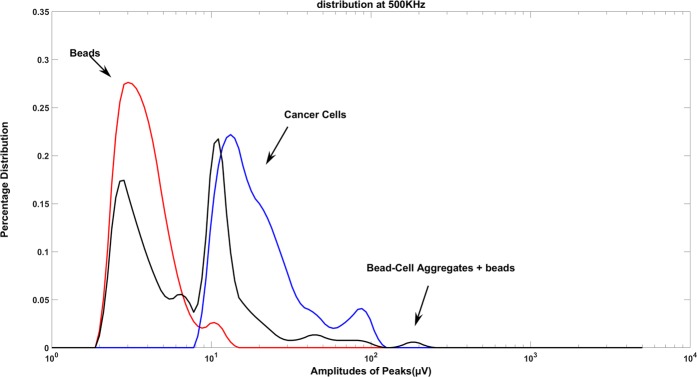
Percentage distribution as a function of peak amplitude for experiments with (1) Pure solution of 2.8 µm beads (red, 300 particles detected). Higher amplitude levels greater than 10 μV are due to beads aggregating, (2) Pure solution of Mantle cells (blue, 300 particles detected), (3) Mixture of bare beads with bead-cell aggregates (black, 200 particles detected). The peaks on the black curve with amplitude greater than 10 μV result from bead-cell aggregates. Frequency and amplitude of the input AC voltage were 500 kHz and 1 V respectively. (p < 0.05 for beads and cell).

As confirmed through visual inspection, bead-cell aggregates show larger diameter compared to both bare cells and bare beads. In the electrical measurements, this manifests itself as larger peak intensity (Fig. 7). In Fig. 8, we plot the distribution of the peak intensity at a frequency of 500 kHz and input AC voltage of 1 V, which is where impedance change of the particle is dominated by the effect of its volume. For the solution of magnetic beads, we see a normal distribution of particles with a mean value of approximately 2 µV. There is a smaller percentage of magnetic beads that exhibit a mean value of approximately 10 µV, which is most likely a result of beads aggregating together non-specifically. The solution consisting of cancer cells primarily exhibits a normal distribution with a mean value of approximately 15 µV. In the same solution there is some variation in particle sizes due to either smaller cells or cells clustered together resulting in a larger peak amplitude. Here, we note that the final mixture, where immune-magnetic separation was performed, consists of a large percentage of bare beads and then a smaller number of bead-cell aggregates. This is because we mix the cells with an overabundance of particles to ensure efficient capture. As a result, we observe a bimodal distribution for this plot (black curve), where the smaller intensity peaks correspond to bare beads, and the larger intensity peaks corresponding to bead-cell aggregates, and the larger sized peaks correspond to large bead-cell aggregates forming.

**Figure 9:**
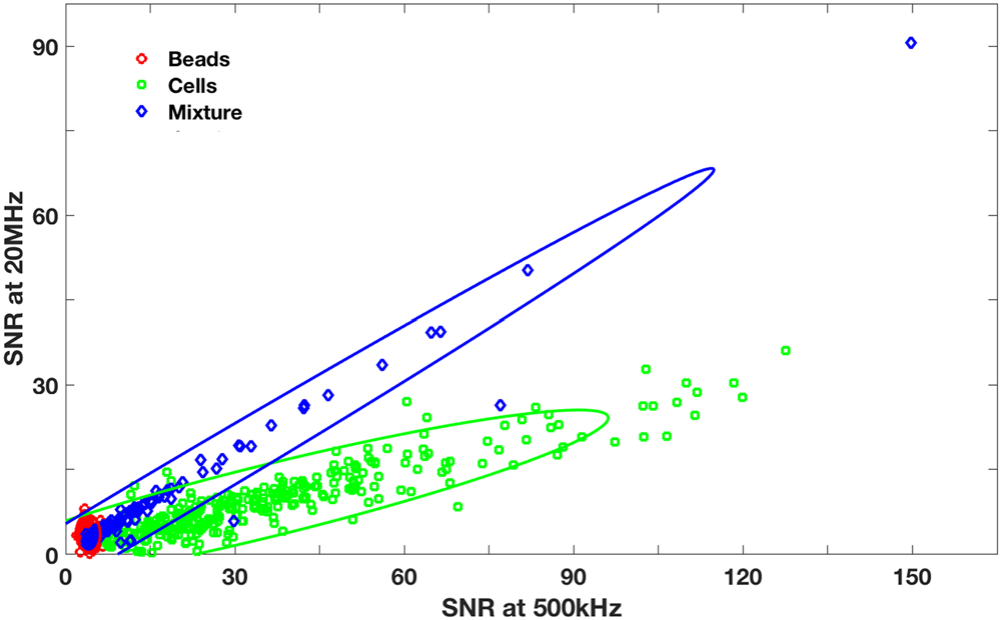
Scatter plots for SNR at 500 kHz and SNR at 20 MHz. Red dots correspond to pure sample of bare beads. Blue dots correspond to the mixture of beads and bead-cell aggregates. Green dots correspond to pure sample of cells. Overlap of the blue and red data sets corresponds to unbound beads.

Observation of the peak intensities as a function of its multiple frequencies at higher dimensions illustrates the ability to differentiate the different particles from each other. The results further demonstrate the ability to classify particle types, when studying the impedance at multiple frequencies simultaneously. The two-dimensional scatter plot for Signal-to-Noise Ratio (SNR) of the peak intensities at 500 kHz and 20 MHz (Fig. 9) are shown. The peak intensities at 500 kHz and 20 MHz for the three data sets (bare bead, bare cells, and bead cell aggregates + beads) are plotted on the scatter plot, each forming distinct clusters. An ellipse is drawn around each data set, the boundaries of which representing the respective standard deviation. The primary goal is to identify the number of cells, which have aggregated with the magnetic beads. Bare magnetic beads (red) form a cluster at the bottom left corner of the x- and y-axis, because of their small relative diameter. Bare cancer cells (green) exhibit larger peak intensities, particularly at f = 500 KHz, yet have lower SNR at f = 20 MHz, thus have a slope of m <1. The mixture (blue) of bare beads with bead-cell aggregates exhibits properties of both particle types. On the one hand, the overall SNR (@ f = 500 KHz) is relatively smaller compared to cells, and a good proportion of them fall within the red ellipse (for bare magnetic beads). The bead-cell aggregates in the mixture have a higher SNR both at f = 500 KHz and 20 MHz compared bare cells and bare beads. Thus, to quantify the number of bead-cell aggregates (to determine the extent of matriptase expression on the cancer cell surface), the particles in the blue ellipse, which have no overlap with the red and green ellipses, will give an accurate count of the bead cell-aggregates in the mixture. Everything in the blue ellipse that does not overlap with the red ellipse represents cancer cells that have been immuno-magnetically separated.

## Conclusions

Our experimental results showed that through the combination of immuno-magnetic cell separation and multi-frequency microfluidic impedance cytometry, we are able to effectively assess the expression of target antigens on the surface of cancer cells. Bare magnetic beads, cancer cells, and bead-cell aggregates exhibit different frequency responses as well as varying voltage peak intensity distributions. We demonstrate this for qualitatively assaying the presence of activated “matriptase” on the surface of cancer cells. The lowest concentration for reliable detection demonstrated in this study was 1.5 million cells/ml. This detection limit is more suitable for detection of dissociated cancer cells obtained from a tissue biopsy as opposed to circulating tumor cells in blood. Further work can be done in developing this novel analytical technique to enable qualitative assessment on matriptase levels. The work shown here, lays the groundwork necessary for developing an integrated biochip with the capability of rapidly assessing whether patients will be responsive to anti-matriptase based cancer therapeutics or not. Future work will be dedicated to isolating circulating tumor cells in blood and determining matriptase levels on captured cells. The combination of this technique along with the use of nanoelectronically barcoded beads^28–30^ can be a potential solution for analysing multiple markers on cell surfaces. Though for our experiment, we focused on matriptase and Mantle cells, we emphasize that this method is applicable to a wide array of markers and cell types.

## Supplementary information

Supplementary information
