# Supplementary material for: Rapid Assessment of Surface Markers on Cancer Cells Using Immuno-Magnetic Separation and Multi-frequency Impedance Cytometry for Targeted Therapy
Source: Sci Rep. 2020 Feb 20;10:3015. doi: 10.1038/s41598-020-57540-7 (PMC7033175; doi:10.1038/s41598-020-57540-7)
Supplement: Supplementary file 1 — Supplementary information [file 41598_2020_57540_MOESM1_ESM.pdf]

Rapid Assessment of Surface Markers on Cancer Cells Using Immuno-Magnetic Separation and Multi-frequency Impedance Cytometry for Targeted Therapy

Zhongtian Lin [a], Siang-Yo Lin [b], Pengfei Xie [a], Chen-Yong Lin [c], Gulam Rather [b], Joseph Bertino [b] and Mehdi Javanmard [a,b,1]

[a] Rutgers University New Brunswick; Department of Electrical and Computer Engineering; 94 Brett Rd New Brunswick, NJ 08854

[b] Cancer Institute of New Jersey; Rutgers University; 195 Little Albany St, New Brunswick, NJ 08901

[c] Georgetown University; School of Medicine; 3900 Reservoir Rd NW, Washington, DC 20007

[1] Email: mehdi.javanmard@rutgers.edu

Supplementary information

As showed in Fig. s1., The gel from the blue background is the original film including the staining of activated matriptase and GPDAH, which is the internal control. The figure we used in paper was cropped from the original without any further manipulation.

Top blot (highlighted with red color, only first four wells belong to MCL cell lines) was probed for M69 (recognizes activated matriptase). Part of the same gel was probed for M24 (lower portion) for different experimental purpose. GAPDH was probed as an input control for both M69 and M24 blots.

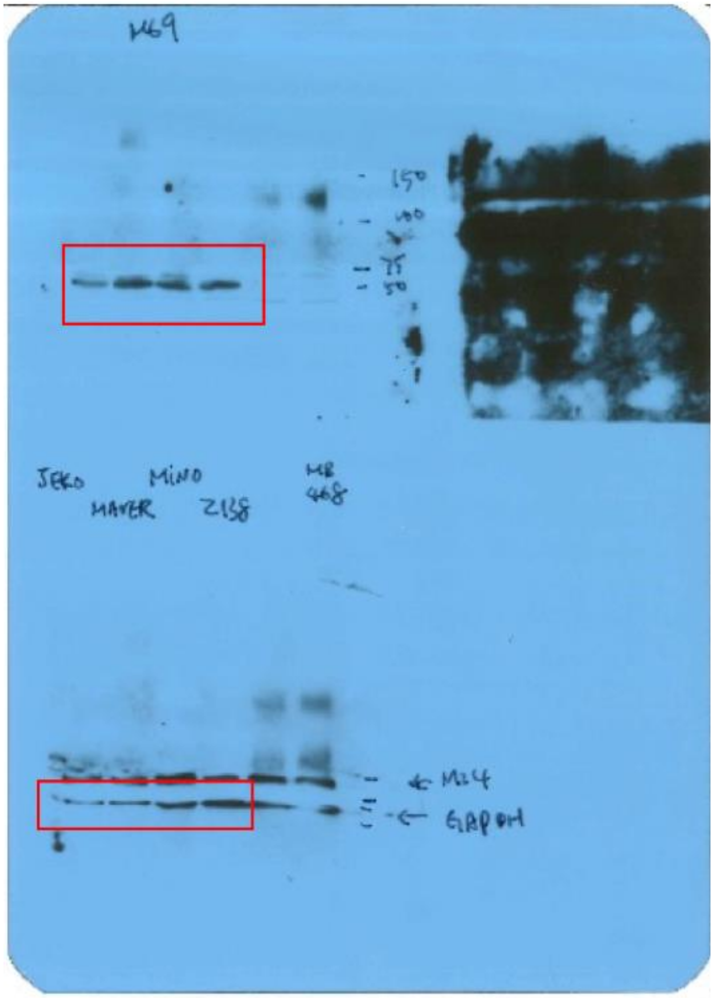

Fig. s1. Western blot analysis of activated matriptase expression in mantle cell lymphoma (MCL) cell lines (JeKo-1, Maver, Mino,and Z138).
